# A Meta-Analysis of Self-Management Interventions in Teaching Daily Living Skills to Autistic Individuals

**DOI:** 10.1007/s10803-024-06355-w

**Published:** 2024-05-06

**Authors:** Orhan Aydin, Mehmet D. Sulu, Ceren Ari-Arat

**Affiliations:** 1 Faculty of Education, Erzincan Binali Yildirim University, 24000 Erzincan, Türkiye; 2 Educational Leadership and Advanced Studies, Northern Kentucky University, Highland Heights, KY 41099 USA; 3 Health Services Vocational School, Bayburt University, Bayburt, Türkiye

**Keywords:** Daily living skills, Autistic individuals, Meta-analysis, Self-management

## Abstract

The current study aimed to conduct a systematic review and meta-analysis of self-management interventions for teaching daily living skills to autistic individuals. This study accessed the corresponding studies by doing a search in six databases. 14 articles and one dissertation met the inclusion criteria. The included studies were first analyzed descriptively and coded according to quality indicators using What Works Clearinghouse (WWC) standards. Second, the effect sizes of the included studies were calculated using two different effect size measures (i.e., Tau-*U* and performance-criteria-based effect size values [PCES]). Third, these analyses were also conducted for generalization and maintenance data. Of 15 studies included in this review, nine met the WWC standards with and without reservations. Tau-*U* analyses were conducted for 14 studies, whereas PCES values were calculated for only eight studies with mastery criteria. The findings indicated that the self-management interventions had a .93 CI_95_ (.80, 1) overall effect size for Tau-*U* with a very large effect. On the other hand, the overall effect size for the PCES values indicated a moderate effect with .99. The weighted effect sizes in generalization and maintenance phases were very large for Tau-*U*; however, moderate to high effects for PCES. Although self-management interventions showed diversity, one of the domains of daily living skills (i.e., community living skills) has not been studied in the field. Notably, among the studies in our review, the last ones are from 2019. Detailed findings from descriptive analyses and two different effect size calculations are discussed, and recommendations for future studies are given.

**Supplementary Information:**

The online version contains supplementary material available at 10.1007/s10803-024-06355-w.

## Introduction

Developing independence in daily living skills such as personal hygiene, meal preparation, and money and time management is important for *all* individuals (Baker et al., 2021; Bal et al., 2015). Acquiring independence at an early age equips one better to excel in aspects necessary for domestic and vocational settings, as well as meaningful social participation (Baker et al., 2021; Pierce & Schreibman, 1994). Autistic individuals, however, often face challenges with independence and depend on others to assist with their everyday tasks (Hume et al., 2009; Smith et al., 2012). Although adult assistance can be supportive for task completion initially, autistic individuals may become dependent on cues from external agents to start tasks or activities, which can lead to an overreliance on external prompts (Martella et al., 2012; Sulu et al., 2023b). Without effective interventions to enhance independence, dependency can persist by limiting career pursuits and involvement in community activities (Hume et al., 2009). Thus, there is a greater focus on finding interventions to shift stimulus control from external agents to autistic individuals (Cooper et al., 2020; Mays & Heflin, 2011; Sulu et al., 2023a).

Self-management interventions are widely recognized for their effectiveness in promoting independence by transitioning the responsibility of behavior management from external agents to the individuals themselves (Cooper et al., 2020; Martella et al., 2012; Sulu et al., 2023a). By personally adopting behavioral strategies, individuals learn to manage their behaviors by identifying, monitoring, and arranging antecedents and consequences for specified target behaviors (Cooper et al., 2020; Martella et al., 2012; Mooney et al., 2005). These interventions have been successfully applied to enhance vocational skills (Beaver et al., 2017), on-task behaviors (Sulu et al., 2023b), negative statements (Dalton et al., 1999), vocal stereotypy (Scalzo et al., 2015), academic outcomes (Wood et al., 2002); across a variety of settings, including schools (e.g., Will & Mason, 2014), homes (Lee et al., 2007), community settings (Lee et al., 2018), and works settings (Beaver et al., 2017). Furthermore, daily living skills such as hygiene (e.g., Duttlinger et al., 2013; Gushanas et al., 2019), food preparation (e.g., Bouck et al., 2014), and cleaning (e.g., Breznack et al., 2012; Lee et al., 2018) have been taught using self-management interventions.

Self-management includes several strategies (e.g., self-evaluation, goal setting, self-charting, self-monitoring). While self-monitoring has primarily been utilized among these interventions (McDougall et al., 2017), combining self-management strategies is not unusual (Cooper et al., 2020; Sulu et al., 2023b). Such integration has been promoted to foster more resilient behavior change (Davis et al., 2014). As an example, Lee et al. (2018) used a self-monitoring intervention that included visual task analysis with pictures and scripts, verbal instructions, in vivo modeling, video self-feedback, and reinforcement in teaching three autistic children to complete dishwashing tasks in home settings independently. The findings of this study showed that all three children enhanced their diswashing skillls and maintained these competencies at mastery level without supervision one week after the intervention concluded. Additionally, technological devices have also been incorporated into self-management interventions. For example, Bouck et al. (2014) focused on enhancing the food preparation skills of three autistic children using technology based (i.e., iPads) versus paper/pencil based self-monitoring interventions through an alternating treatment design. The study measured indepenedence in completing recipe steps, the number of prompts required, and total duration of each recipe. The findings revealed that all three participants were more independent and completed recipe steps using the iPad app in less time than when using paper/pencil. Despite the use of different intervention components, the authors recommended that self-management interventions effectively increase the daily living skills of autistic individuals and advocate for their broader application in future research (Bouck et al., 2014; Lee et al., 2018).

Given the importance of daily living skills in promoting the independence of autistic individuals, the efficacy of self-management interventions to achieve this, and the wide range of variables included in the implementation of self-management interventions, it is important to assess the overall effects of these interventions. Fortunately, several meta-analyses and systematic reviews on self-management interventions have been conducted in a broad context. However, there are no specific systematic reviews or meta-analyses that exclusively focus on evaluating the effectiveness of self-management interventions in improving the daily living skills of autistic individuals, to the best of our knowledge. This gap encompasses other self-management strategies, including self-monitoring (e.g., Sulu et al., 2023a) and self-reinforcement (e.g., Brown et al., 2018). Although not focused on daily living skills and autistic individuals, existing reviews and meta-analyses, including those by Carr et al. (2014), McDougall et al. (2017), and (Yucesoy-Ozkan & Sonmez, 2011), may touch upon aspects relevant to our study. Therefore, it may be important to discuss the findings of these studies along with their limitations.

Yucesoy-Ozkan and Sonmez (2011) conducted a meta-analysis to investigate the effect of self-management interventions for individuals with developmental disabilities and included a total of 40 single-case experimental designs (SCEDs) studies published in peer-reviewed journals between 1999 and 2008. In their review; autistic individuals were one-third of the sample, and daily living skills were not mentioned. As for their analysis, Yucesoy-Ozkan & Sonmez used the percentage of nonoverlapping data (PND) and the percentage of zero data (PZD; Scruggs et al., 1987). Despite some key insights into the quality and the magnitude of self-management interventions from that meta-analysis, the review did not make the quality evaluations of the research to verify methodological rigor and experimental control in the included studies. However, conducting a quality assessment using rubrics such as suggested by Kratochwill et al., (2013; What Works Clearinghouse [WWC], 2022) ensures that conclusions are based only on high-quality studies. Another meta-analysis conducted by Carr et al. (2014) investigated self-management interventions on autistic students, which yielded a total of 23 SCEDs studies published until 2012. Although the Carr et al. (2014) study is more relevant to the current review, it included only one study (Pierce & Schreibman, 1994) on daily living skills. As for their analysis, they used WWC standards to investigate the quality of the studies before running the meta-analysis. Based on their analysis of PND for 23 studies, Carr et al. (2014) suggest that self-management interventions are, in particular, effective in improving the social and academic skills of autistic students.

A more recent meta-analysis conducted by McDougall et al. (2017) was for self-management studies conducted in inclusive classrooms, so that they included 29 SCEDs studies published in peer-reviewed journals from 2005 to 2014. Although about half of the 29 studies (*n* = 14) included autistic individuals, they did not focus on daily living skills but were related to on-task, social, and academic skills in the context of inclusion. In addition, this research had some methodological limitations. Similar to the (Yucesoy-Ozkan & Sonmez, 2011) study, no quality rubric was conducted. Another limitation is that the effect sizes in their meta-analysis were measured with the percentage of nonoverlapping data (PND), percentage of all nonoverlapping data (PAND), Phi, and Tau-*U*, which are nonoverlap-based methods.

In addition to not focusing on daily living skills of autistic individuals, the last search across the three studies dates back ten years or more. Furthermore, the meta-analysis tools used in these reviews (i.e., nonoverlap-based methods) have been criticized for not being able to reveal the true effect size due to based on data overlapping and not considering data trend (Aydin & Tanious, 2022; Wolery et al., 2010). Moreover, none of these studies attempted to review gray literature, such as theses and dissertations. However, recent findings indicate that relying solely on peer-reviewed publications for a meta-analysis can introduce a publication bias (Gage et al., 2017; Ploanin et al., 2016). Therefore, the inclusion of gray in meta-analyses is recommended.

Beyond these limitations, the previous literature lacked an investigation into generalization and maintenance of the included studies. The previous meta-analyses included only A (baseline) and B (intervention) conditions without running a systematic effect size analysis in generalization and maintenance. On the other hand, evaluating the generalization and maintenance of self-management interventions is crucial (Cooper et al., 2020; Sulu et al., 2023a; Wood et al., 2002), given that autistic individuals ought to have the ability to apply newly acquired daily living skills to new and similar stimuli that were not part of the initial training environments (Sulu et al., 2023a, 2023b). Therefore, investigation of the self-management interventions in teaching daily living skills to autistic individuals and the use of different effect size logics for analyses of intervention effects (e.g., Tau-*U* and performance criteria based effect size [PCES]) that overcome the mentioned limitations would give a more comprehensive understanding of these interventions to the audience and also may assure more reliable and valid results of intervention effects about this topic.

Given the importance of daily living skills for autistic individuals, the diversity of the self-management interventions to improve such skills, the lack of investigation for daily living skills, and the limitations of the previous literature to investigate the aggregation of the literature, including generalization and maintenance; there is a need for a new systematic review and meta-analysis study to catch out the general trend related to this topic. Therefore, the current study will contribute to the literature by aggregating and comparing the results of self-management studies in teaching daily living skills to autistic individuals. In these regards, the specific research questions are as follows:

1. What are the descriptive characteristics of self-management interventions in teaching daily living skills to autistic individuals?
2. What is the overall quality of self-management interventions in improving the daily living skills of autistic individuals based on WWC standards for single-case experimental designs?
3. What is the magnitude of the effect of self-management interventions in acquiring daily living skills of autistic individuals?
4. What is the magnitude of the effect of self-management interventions in teaching daily living skills to autistic individuals on the generalization and maintenance data?

## Method

### Search Procedure

Before starting the review process, the first author formed an initial search protocol and shared it with all stakeholders at a Zoom meeting (see this protocol in the supplemental materials). In parallel with this protocol, the search included only SCEDs studies published in English and peer-reviewed journals. Given that autism had been first categorized as a distinct diagnostic category by the American Psychiatric Association (APA) in the 1980 edition of the Diagnostic and Statistical Manual of Mental Disorders- III (DSM-III), the search included studies beginning between 1980 January and 2023 August. The first search was conducted in ERIC, Academic Seach Ultimate, ScienceDirect, PsycNET, and Scopus databases by accessing them through the libraries of the universities where the first and third authors were employed. Key terms were broken down into three categories (a) self-management strategies, (b) daily living skills, and (c) autism. Within each group, keywords are combined using “OR”; across groups, keywords are combined using “AND". The keywords included for each category, respectively: *Self-management, self-regulation, self-regulate, self-monitoring, self-recording, self-reinforcement, self-evaluation, self-advocacy, self-observation, self-instruction, empowerment, self-determination*, *self-control* (AND) Autis*, asperger’s syndrome, PDD-NOS* (AND) *daily living skills*, *self-care*, *self-help*, *life-skills*, *functional skills*, *leisure skills*, *recreation skills*, *living skills*, *adaptive living*, *activities of daily living*, independen* living, independen* skill, *practical skill*. The first search was driven between 13 and 25 August and once per database independently by the first and third authors. Upon the reviewer’s advice, the second search, similar to the first search procedure, was also conducted in ProQuest and Thesis Global database to capture the corresponding gray literature on February 19, 2024. After organizing the search results via Zotero, both authors transferred their first and second search documents to the Rayyan system. Rayyan is a system of the systematic review process that makes it easy to collaborate with the research team and provides reliability evaluations more quickly. 2,072 articles and 67 dissertations/theses were identified in the initial search. After removing the duplicates (*n* = 387), 1,752 studies were transferred to Rayyan to be evaluated using inclusion and exclusion criteria (see Figure S1 in the supplementary materials).

### Inclusion and Exclusion Criteria

To include articles in the present study, we considered the following inclusion criteria: (a) publishing in a peer-reviewed journal in English, (b) including at least one participant with a diagnosis of autism; however, if one or some of the participants are a part of several other participants who did not have an autism diagnosis, only data of the autistic participants are included, (c) using SCEDs as the research method, (d) having a graphical representation of data in a line format, (e) targeting to improve the daily living skills, and (f) using a self-management strategy as an independent variable. We excluded studies that did not meet the abovementioned inclusion criteria for the present review.

According to inclusion criteria, 1,685 articles and 67 dissertations/theses were evaluated, reviewing the titles and abstracts of the studies on Rayyan, if required, as well as their full texts by accessing them. After evaluations, nine articles and one dissertation were included; however, backward and forward searches were also conducted to determine additional possible studies. The backward search, i.e., reference lists of the included studies and the related review studies, yielded four additional articles to be included. On the other hand, the forward search, i.e., the Google Scholar cited lists of the included articles, found one additional article. A total of 15 studies (14 articles and one dissertation) were included for descriptive codings and evaluations in terms of WWC standards.

## Descriptive Codings

We reviewed the included 15 studies according to the following descriptive parameters: (a) participant characteristics, including gender, age, and diagnosis; (b) practitioner; (c) dependent variables (DVs); (d) independent variables (IVs) and also components of IVs; (e) research design and mastery criteria; (f) interobserver agreement and treatment fidelity; and (g) social validity, generalization, and maintenance. The first and third authors coded all studies according to these parameters.

## Quality Assessment According to WWC Standards

We evaluated the included studies based on the WWC standards (Kratochwill et al., 2013). We followed up on the protocol for WWC standards, which was adapted by Maggin et al. (2013) and revised by Hong et al. (2016). This protocol covers the assessments of design standards (DSs) for SCEDs, including (a) systematic manipulation of independent variables showing DS1, (b) interrater reliability showing DSs 2A, 2B, and 2C, (c) three demonstrations of the intervention effect showing DS3, and (d) whether an adequate number of data points is provided for each condition showing DS4. We present detailed explanations about the ratings of the DSs in Table S1 in the supplemental materials; we also refer readers to review the studies which were conducted by Maggin et al. (2013), Hong et al. (2016), Neely et al. (2016), Neely et al. (2018), Sulu et al. (2023a), and Sulu et al. (2024). According to the protocol, we evaluated the articles as “meet standards” with a “2” when all standards were met with the highest possible score. If one of the DSs was rated with a “1” where the maximum score was “2,” the article was labeled as “meet standards with reservations.” On the other hand, a score of “0” indicated that at least one of the DSs was coded with a “0” and did not meet standards. The first and third authors rated all included studies according to the WWC standards. These studies also were evaluated quality assessment for generalization and maintenance phases according to WWC standards by following up on Neely et al. (2018) adapted rubric (see Table S2 in the supplemental materials).

## Data Extraction

We used PlotDigitizer, a free, reliable, and valid software program for digitizing data (Aydin & Yassikaya, 2022; Huwaldt, 2020), to extract raw data from single-case graphs. Data on the baseline (A), intervention (B), generalization (G), and maintenance (M) phases were digitized. After each related phase on the graphs was digitized, extracted raw data were transferred to the Numbers documents (MacOS files). As suggested by Aydin and Yassikaya (2022), for more validity and reliability in effect size measures, they were rounded to the nearest integer value by looking at each graph. The first and third authors extracted raw data from all related graphs (*n* = 60) derived from studies (*n* = 14). Note that the included dissertation was excluded from this and further processes due to the lack of comparable baseline data. After digitizing the corresponding graphs in 14 studies, effect sizes were calculated using the raw data of the included phases.

## Effect Size Measurements

We calculated Tau-*U* (Parker et al., 2011) and performance criteria-based effect size (PCES; Aydin & Tanious, 2022) values to obtain the effect sizes of interventions in the included 14 studies. We chose them because both effect sizes can control the baseline trend, which may disrupt the calculation of the actual treatment effect. In addition, since they contain different measurement logics, they allow us to calculate the magnitude of the intervention effects with different aspects. Tau-*U* is a nonoverlap-based method that calculates the effect size by taking into account the overlap among data on graphs. On the other hand, PCES is a mean-based method that calculates intervention effect sizes, considering the skill acquisition levels through treatment. In Tau-*U* calculations, it is recommended to control the baseline trend if there is a contrast of 0.40 or above among baseline data (i.e., Tau-*U*_*Bc*_ [Tau-U baseline correction] should be estimated; Brossart et al., 2018). In PCES calculations, Aydin and Tanious (2022) recommended controlling baseline trends via the split middle method if there is a visually noticeable linear trend according to the split middle method.

In the present study, we obtained effect size values by comparing A (baseline)—B (intervention), A (baseline)—G (generalization), and A (baseline)—M (maintenance) for the graphs that were included in further analysis. PCES or PCES_trend_ values were calculated by hand according to the formulae suggested by Aydin and Tanious (2022), whereas Tau-*U* or Tau-*U*_*Bc*_ values were obtained through a web-based calculation engine (http://singlecaseresearch.org/calculators/tau-u). Moreover, weighted average Tau-*U*/Tau-*U*_Bc_ and PCES/PCES_trend_ values were calculated to determine the overall effect of the studies and moderators. The web-based calculation engine was used for weighted average Tau-*U* values*,* while the following formula was used by hand for weighted average PCES values for individual studies and overall results of the included studies:

$$PCE{S}_{weighted}=\frac{{\sum }_{i=1}^{N}{k}_{i}PCE{S}_{i}}{{\sum }_{i=1}^{N}{k}_{i}}$$

where N = number of the included AB phases in the study for *individual study results*; number of the included studies for *overall results*; k_i_ = number of data in i. AB phase for *individual study results*; number of PCES values in i. study for *overall results* (i.e., number of AB phase included for PCES calculations in i. study), PCES_i_ = PCES value for i. AB phase for *individual study results*; weighted average PCES value in i. study for *overall results*. Note that PCES values were obtained only in the studies with mastery criteria because PCES can be calculated according to a performance criterion set in the articles (cf. Aydin & Tanious, 2022).

Tau-*U* values also give confidence intervals (CI); hence, they allow us to see how Tau-*U* values ranged within CI with 95% (CI_95_). The closer the range with CI_95_ of the Tau-*U* value is, the more precise the calculated effect size is; otherwise, the precision is decreased. In this regard, we also depicted a forest plot graph for Tau-*U* values with CI_95_. A forest plot allows the results of studies to be reported individually and/or combined by showing the precision and size of the intervention effect (Cooper et al., 2020; Sulu et al., 2023b). The first and third authors calculated all intervention effect sizes in the included studies following the instructions mentioned above. The first author also created the forest plot graph and calculated the weighted PCES values.

Upon interpretations of effect sizes calculated for interventions in the included studies, we followed up on the following benchmarks. Vannest and Ninci (2015) suggested that Tau-*U* values can be interpreted as small for $\le$ 0.20, moderate/medium for [0.21; 0.60], large for [0.61; 0.80], and very large for 0.81 or above. For PCES values, Aydin and Tanious (2022) offered to interpret PCES values separately depending on whether PCES_trend_ is calculated. In the present study, we interpreted weighted average PCES values in a study considering the PCES type (PCES without trend or PCES_trend_) most calculated in that study. PCES is ineffective for $\le$0.39, very small effect for [0.40; 0.60], small effect for [0.61; 0.84], moderate effect for [0.85; 1.01], effective for [1.02; 1.16], high effect for $\ge$ 1.17; on the other hand, PCES_trend_ is interpreted ineffective for $\le$ 0.20, very small effect for [0.21; 0.39], small effect for [0.40; 0.67], moderate effect for [0.68; 0.91], effective for [0.92; 1.11], high effect for $\ge$ 1.22.

## Intercoder Reliability

We conducted the reliability analyses for the following six conditions: (a) search procedure, (b) inclusion and exclusion process, (c) descriptive codings, (d) assessment according to WWC standards for SCEDs, (e) data extraction, and (f) effect size calculations. All of the intercoder reliability analyses were calculated as a point-by-point method that was used to determine the percentage of intercoder agreements by dividing the number of agreements by the total number of agreements plus disagreements and multiplying by 100. The first and third authors conducted search procedures independently on the databases, and the reliability was found to be 100%. In the inclusion and exclusion process, the third author reviewed all studies, whereas the first author reviewed approximately 42% (*n* = 741) of the studies transferred into Rayyan. The intercoder reliability between the first and third authors for this stage was found to be 98.6%. Disagreements between two coders were resolved, and 100% consistency was achieved.

As for descriptive codings, the first and third authors coded all included studies (*n* = 15). The reliability percentage was calculated as 97.8% for these codings. Inconsistencies between coders were discussed, and 100% consistency was obtained. In the assessment according to WWC standards, the first and third authors coded all included studies in terms of WWC standards, and the intercoder reliability was calculated as 94.4%; however, all inconsistencies were resolved after discussions. Given data extraction reliability, the first and third authors digitized all related graphs (*n* = 60) in 14 studies, and the reliability analysis resulted in about 100% when accepting between two coders ± 1 unit deviations in data digitizations as a consistency. As for the reliability analysis of effect size calculations, the first and third authors independently calculated all effect size values of the graphs in the included studies. The reliability was 98.1% for Tau-*U* values but 98.8% for PCES calculations. Minimal discrepancies for Tau-*U* and PCES were cleared up by recalculations.

## Results

### Descriptive Analysis

All the included studies (*n* = 15) were analyzed based on the participant and the intervention characteristics. The findings are depicted in Table S3 in the supplementary materials.

**Participant Characteristics:** Of the 15, a total of 31 autistic individuals were involved. While all the participants had solely a diagnosis of autism in 67% (*n* = 10) of the studies, 33% (*n* = 5) included autistic individuals and also other developmental disabilities. Given that the data were aggregated for the autistic individuals in these studies, one of the two (Copeland & Hughes, 2000), one of the three (Stokes et al., 2004), one of the four (Duttlinger et al., 2013; Mechling & Stephens, 2009), and one of the five (Gushanas et al., 2019) participants were included for the present review. A total of 22 males (71%; e.g., Lee et al., 2018) and nine autistic females (29%; e.g., Mays & Heflin, 2011) were derived across the studies. The participants’ ages ranged from 6 to 37, with an average age of 14. The majority of participants had autism (94%, *n* = 29; e.g., Bouck et al., 2014; Lee et al., 2018; Mays & Heflin, 2011), the remaining two participants (6%, *n* = 2; Yakubova & Taber-Doughty, 2012) was diagnosed with high-functioning autism. About 61% (*n* = 19) of the autistic participants also had intellectual disabilities in degrees with severe (13%, *n* = 4; e.g., Bouck et al., 2014), moderate (29%, *n* = 9; e.g., Mays & Heflin, 2011), and mild (19%, *n* = 6; e.g., Yakubova & Taber-Doughty, 2012).

**Methodological Characteristics:** The methodological characteristics of the studies included (a) settings and instructional arrangement, (b) practitioners, (c) dependent variables, (d) independent variables and components of IV(s), (e) research design, (f) mastery criteria, (g) interobserver agreement and treatment fidelity, and (h) social validity, (i) generalization and maintenance.

### Setting(s) and Instructional Arrangement

Settings are different per a study, including as follows: school living center and the teacher workroom; classroom; bakery simulation in the classroom; faculty dining room at the participants’ high school; participants’ self-contained classroom, the hall immediately adjacent to the classroom and the bathroom on this hallway; a clinic room at the Autism Center of a University; a child care center classroom-community locations, university classrooms and office; home; kitchen of the house and autism center; restroom; kitchen; home kitchen, school and restaurant; clinic room; bathroom; classroom that included a full kitchen, dining area and a bathroom. Of all the studies, the instruction arrangement was one-to-one (see Table S3 in the supplementary materials).

### Practitioner

The interventions were conducted by a variety of practitioners. Researchers served as practitioners in about half of the included studies (47%, *n* = 7; e.g., Gushanas et al., 2019; Mechling & Stephens, 2009). Teachers took part in one-fifth of the studies (20%, *n* = 3; e.g., Mays & Heflin, 2011). In the remaining 33% (*n* = 6) of the included studies, instructor (Lee et al., 2018), para-professional (Parker & Kamps, 2011), therapist (Pierce & Schreibman, 1994), parent (Lee et al., 2007), clinician (Engstrom, 2019), and educational assistant (Copeland & Hughes, 2000) participated as a practitioner. Note that Bereznak et al. (2012) employed two practitioners together, including a researcher and a teacher.

### Dependent Variables

Dependent variables included a variety of skills such as meeting personal skills, including preparing food, cooking, making drinks, buying groceries, and restaurant activities (47%, *n* = 7; e.g., Bouck et al., 2014; Parker & Kamps, 2011); home management skills, including using laundry, cleaning mirrors/sinks/floors, washing dishes, arranging a table, cleaning under and over the table, and making a bed as home management skills (40%, *n* = 6; e.g., Copeland & Hughes, 2000; Engstrom, 2019; Yakubova & Taber-Doughty, 2012); personal hygiene skills, including brushing teeth and washing hands (33%, *n* = 5; e.g., Duttlinger et al., 2013; Gushanas et al., 2019). Note that several studies (e.g., Bereznak et al., 2012; Engstrom, 2019) included more than one dependent variable.

### Independent Variables and Components of them

The majority of studies (73%, *n* = 11; e.g., Bouck et. al., 2014; Engstrom, 2019; Gushanas et al., 2019) used self-monitoring as an independent variable. Self-instruction (20%, *n* = 3; e.g., Mechling & Stephens, 2009), self-recording (13%, *n* = 2; Lee et. al., 2018; Parker & Kamps, 2011), self-reinforcement (13%, *n* = 2; Lee et al., 2007; Pierce & Schreibman, 1994), self-selection (7%, *n* = 1; Cheung et al., 2016) and self-evaluation (7%, *n* = 1; Stokes et al., 2004) were used as an independent variable in a small number of studies. Note that several studies (e.g., Cheung et al., 2016; Lee et al., 2007) included more than one independent variable. Among the studies that used self-monitoring (*n* = 11), self-monitoring was used alone in about 45% (*n* = 5; e.g., Bouck et al., 2014; Gushanas et al., 2019); on the other hand, self-monitoring was used together with another instruction, such as self-selection, self-reinforcement, and self-evaluation, in about 55% (*n* = 6; e.g., Cheung et al., 2016; Parker & Kamps, 2011).

Upon review of the components of the independent variables, written documents were used in 67% of the studies (*n* = 10; e.g., checklist, task analysis, recording form); however, visual aids were used in 60% (*n* = 9, e.g., videos, activity schedules, picture prompt booklet, picture-based cookbook, video modeling clips). On the other hand, auditory materials (13%, *n* = 2; e.g., cassette tape player, DVD player) and error correction (7%, *n* = 1; i.e., least to most prompting procedure) were used in a small number of studies. Almost all studies included more than one component for the independent variable.

### Research Design

More than half of the studies used multiple baseline/probe designs (60%, n = 9; e.g., Mays & Heflin, 2011; Yakubova & Taber-Doughty, 2012). One-fifth of the studies employed reversal/withdrawal designs (n = 3; Duttlinger et al., 2013; Lee et al., 2007; Stokes et al., 2004). About 13% of the studies used alternating treatment design (*n* = 2; Bouck et al., 2014; Mechling & Stephens, 2009); however, only one study (7%; Engstrom, 2019) employed MBD with reversal design.

### Mastery Criteria

More than half of the studies (60%, n = 9) set up mastery criteria. Among these studies, more than half (55%, n = 5; e.g., Lee et al., 2018; Pierce & Schreibman, 1994) set it at 100%. Bereznak et al. (2012), however, shifted the initial mastery criteria from 100 to 90% based on a clinical decision during the intervention. One study (11%; Duttlinger et al., 2013) set at %80, one other study (11%; Parker & Kamps, 2011) determined mastery criteria as the independent compilation of half of the skills listed in the task analysis and another study (11%; Engstrom, 2019) set it as 75%.

### Reliability

Almost all studies (93%, n = 14; e.g., Mays & Heflin, 2011; Mechling & Stephens, 2009) provided interobserver agreement (IOA) for the dependent variable. Only Lee et al. (2007) did not provide IOA for dependent variables. Among those that collected IOA, only Copeland and Hughes (2000) collected less than 20%. As for obtaining treatment fidelity data across the included studies, only 47% of them (n = 7; e.g., Bouck et al., 2014; Engstrom, 2019; Duttlinger et al., 2013) collected treatment fidelity data.

### Social Validity

Of the 15 studies, about half (47%, n = 7; e.g., Cheung et al., 2016; Duttlinger et al., 2013) collected social validity data from various agents. All social validity data were collected through subjective assessments. Among those that obtained social validity data, 43% (n = 3; e.g., Gushanas et al., 2019) were collected from the participants, and 43% (n = 3; e.g., Yakubova & Taber-Doughty, 2012) were collected from teachers. The social validity was also obtained from external agents (Cheung et al., 2016), parents (Lee et al., 2018), paraprofessionals (Duttlinger et al., 2013), and speech-language therapists (Duttlinger et al., 2013). Three studies that obtained social validity (43%, e.g., Duttlinger et al., 2013; Yakubova & Taber-Doughty, 2012) collected it from more than one agent.

### Generalization/Maintenance

About one-third of the included studies (33% n = 5; e.g., Stokes et al., 2004; Yakubova & Taber-Doughty, 2012) collected generalization data. Generalization data were collected across settings in these studies. Maintenance data were collected from 47% of the included studies (n = 7; e.g., Cheung et al., 2016). The maintenance length varied between 1 week and 18 months. Of the seven studies that collected maintenance data, one study (14%; Engstrom, 2019) collected one data point, one study (14%; Cheung et al., 2016) collected 10 data points, and five studies (71%) collected two data points for each participant (e.g., Bouck et al., 2014; Pierce & Schreibman, 1994).

#### Results of Quality Assessment According to WWC Standards

The included 15 studies were also evaluated in terms of WWC standards. Table S4 in the supplementary materials shows the results of the WWC assessments. Only one study (7% of 15 studies; Yakubova & Taber-Doughty, 2012) met standards without reservations, while eight studies (53%; e.g., Duttlinger et al., 2013; Lee et al., 2018) met standards with reservations. Contrary, six studies (40%; e.g., Cheung et al., 2016; Engstrom, 2019) did not meet design standards, although DS1 was met across all articles. Within the articles that did not meet WWC standards with or without reservations, five studies (33%; e.g., Mechling & Stephens, 2009; Stokes et al., 2014) failed to meet only DS3, while one study (7%; Lee et al., 2007) failed to meet DSs 2A, 2B, 2C and 4.

Considering Neely et al.’s (2018) WWC rubric, we also evaluated the generalization (G) and/or maintenance (M) phases of the nine studies (60%) with G/M data. Table S5 in the supplementary materials shows the evaluations of G and M phases. Depending on their presence in the single-case graphs of the nine studies, either G or M or both were evaluated. Two studies (22%; Duttlinger et al., 2013; Yakubova and Taber-Douhty, 2012) were evaluated for only G phases, while the four (45%; e.g., Bouck et al., 2014-iPad; Lee et al., 2018) were assessed for only M phases. Three studies (33%; e.g., Pierce & Schreibman, 1994) were evaluated for both phases. Maintenance phases in two studies (Lee et al., 2018; Pierce & Schreibman, 1994) met WWC standards adapted by Neely et al. (2018), the five studies’ maintenance phases (e.g., Bouck et al., 2014; Engstrom, 2019) did not meet WWC standards, although they have one or two data. The generalization phases in three studies met WWC standards; however, the other two studies’ generalization phases (Cheung et al., 2016 and Stokes et al., 2004) did not meet WWC. The generalization phases of Yakubova and Taber-Doughty (2012) were coded in the highest scores according to Neely et al.’s rubric.

#### Results of Effect Size Measurements

**Fig. 1:**
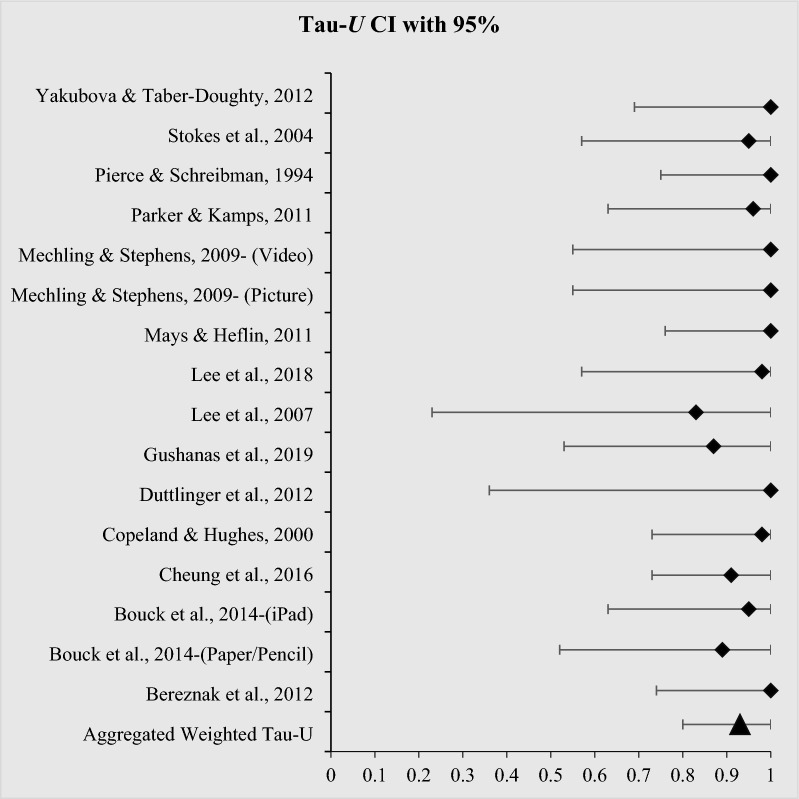
*Forest Plot for Tau-U values with CI*_*95*_* of the included articles*

Tau-*U* values were obtained in all studies (except for Engstrom[, 2019] due to the lack of comparable baseline data); however, PCES values were calculated for eight studies (57% of 14 studies) due to setting mastery criteria in these articles. Table 1 shows the results of Tau-*U* calculations. Tau-*U* was calculated for 60 AB phases derived from 14 studies. The ranges of weighted Tau-*U* values for individual studies were 0.83 to 1. The interventions in all studies had a very large effect size over the dependent variables. In parallel with these findings, aggregated weighted Tau-*U* was calculated to be 0.93 and interpreted to have a very large effect size. Due to obtaining CI_95_ for Tau-*U* values, Fig. 1 also shows Tau-*U* values with CI_95_ ranges in a forest plot graph. Although the aggregated weighted Tau-*U* value (0.93; CI_95_ [0.80; 1]) indicates a more precise effect size, some individual studies, particularly for Duttlinger et al., 2013, Bouck et al., 2014-pencil/paper and Lee et al., 2007, had a more large CI_95_ range and thus, the precise of their weighted effect sizes may be weak (see Fig. 1).

**Table 1:** Tau-U effect size calculations and interpretations of 14 articles

Studies	Number of AB contrasts	Total pairs	Number of Tau-*U*_*Bc*_ calculations (If Tau-*U*_*A*_ $\ge$.40)	Number of Tau-*U* calculations	Weighted Tau-U with 95% CI	Interpretation
Bereznak et al., 2012	9	228	0	9	1 (.74; 1)	Very large
Bouck et al., 2014-(Paper/Pencil)	3	134	0	3	.89 (.52; 1)	Very large
Bouck et al., 2014-(iPad)	3	239	0	3	.95 (.63; 1)	Very large
*Cheung et al., 2016	2	82	0	2	.91 (.44; 1)	Very large
*Copeland & Hughes, 2000	2	924	0	2	.98 (.73; 1)	Very large
Duttlinger et al., 2013	2	27	0	2	1 (.36; 1)	Very large
Gushanas et al., 2019	1	294	0	1	.87 (.53; 1)	Very large
*Lee et al., 2007	2	43	0	2	.83 (.20; 1)	Very large
Lee et al., 2018	3	149	1	2	.98 (.57; 1)	Very large
Mays & Heflin, 2011	7	318	0	7	1 (.76; 1)	Very large
*Mechling & Stephens, 2009- (Picture based)	3	81	0	3	1 (.55; 1)	Very large
*Mechling & Stephens, 2009- (Video recipes)	3	81	0	3	1 (.55; 1)	Very large
Parker & Kamps, 2011	4	266	1	3	.96 (.63; 1)	Very large
Pierce & Schreibman, 1994	9	249	0	9	1 (.75; 1)	Very large
*Stokes et al., 2004	1	588	0	1	.95 (.57; 1)	Very large
Yakubova & Taber-Doughty, 2012	6	150	0	6	1 (.69; 1)	Very large
**Total/ Aggregated Weighted Tau-*U***	60	3853	2	58	.93 (.80; 1)	Very large

Asterisks show the studies that do not meet WWC standards with or without reservations

Table 2 shows PCES values calculated for 32 AB phases of eight studies. The ranges of weighted PCES values for individual studies were 0.72 to 1.55. Four studies had a moderate effect, while two had a high effect. On the other hand, the aggregated weighted PCES value was calculated as 0.99 and interpreted to have a moderate effect on the overall results. Note that Tau-*U*_Bc_ was calculated for only two (3%) of 60 AB phases (see Table 1); however, PCES_trend_ was calculated for seven (22%) of 32 AB phases (see Table 2). PCES values were interpreted considering PCES cutoff scores (not PCES_trend_ cutoff scores) since most of the calculations were PCES values without trend, as explained in the method section.

**Table 2:** PCES calculations and interpretations of eight articles setting a mastery criterion

Studies	Number of AB phases	MC	Number of PCES_trend_ calculations (Determined via split middle)	Number of PCES calculations	Weighted PCES	Interpretation
Bereznak et al., 2012	9	90%	4	5	.89	Moderate
*Cheung et al., 2016	2	80%	0	2	1.06	Effective
*Copeland & Hughes, 2000	2	100%	0	2	.72	Small effect
Duttlinger et al., 2013	2	80%	0	2	1.55	High effect
Lee et al., 2018	3	100%	1	2	.92	Moderate
Parker & Kamps, 2011	4	Half of steps	1	3	1.17	High effect
Pierce & Schreibman, 1994	9	100%	1	8	.97	Moderate
*Stokes et al., 2004	1	100%	0	1	.93	Moderate
**Total/Aggregated weighted PCES**	32	-	7	25	.99	Moderate

Asterisks show the studies that do not meet WWC standards with or without reservations

We also obtained the weighted effect sizes for the G/M phases in the eight studies (57% of the 14 studies), as seen in Table S5 in the supplementary materials. For generalization, Tau-*U*_weighted_ values were found to be constant as 1 with wide CI_95_ ranges for four studies with G phases and 0.92 with a wide CI_95_ for one study, while PCES_weighted_ values were found to be 0.94 to 1.6 for four studies with mastery criteria. For M data, Tau-*U*_weighted_ values ranged from 0.94 to 1 with wide CI_95_ ranges for six studies with M phases; however, PCES_weighted_ values were found to be 0.89 to 1.25 for four studies with mastery criteria. We interpret these values as very effective for Tau-*U* and having a moderate to high effect with respect to PCES, considering explanations about cutoff scores in the method section.

## Discussion

The present review aims to (a) define the characteristics of self-management interventions in teaching daily living skills descriptively, (b) investigate the quality of these interventions by using WWC standards, (c) calculate the magnitude of the effect of the interventions through two different effect size analysis tools (i.e., Tau-*U*, PCES), and (d) specifically analyze their impact in the generalization and maintenance phases. In this regard, this study reviewed the related literature from 1980 to 2023 (for gray literature to 2024). Although 15 studies were included in the further analyses, the most current research (*n* = 2) were published in 2019. While personal and domestic skills were assessed across the included studies, none of the included studies investigated the efficacy of self-management interventions for community living skills. Self-monitoring appeared to be the most commonly used strategy among other self-management interventions to teach daily living skills, yet it was often used in isolation. The studies were predominantly implemented by researchers without incorporating caregivers and teachers in the intervention process—a factor crucial for enhancing the generalization and maintenance of newly acquired skills. Additionally, the overall quality of included studies was deemed weak based on WWC standards, and there was a lack of a comprehensive social validity assessment and clear definition of mastery criterion. Despite these limitations, the meta-analysis of this systematic review suggests that self-management interventions appear to be *promising* to foster a relatively wide range of daily living skills of autistic individuals in varied settings. A detailed analysis of these findings is discussed as follows.

First, the dependent variables included in the studies had some limitations, particularly in the categorization of daily living skills. According to Sparrow et al. (2005), these skills are divided into three groups including personal (e.g., eating, dressing, hygiene, cleaning), domestic (e.g., housekeeping, meal preparation), and community living skills (e.g., time and money management, using transportation). In their recent review of the use of technological devices to teach daily living skills, Hrabal et al. (2023) noted an absence of studies focusing on community living skills for autistic individuals. Although there were studies to teach personal and domestic skills, these daily living skill components included a limited range of target behaviors; for instance, dressing was not studied in any of the reviewed studies (Hrbal et al., 2023). Our findings aligned with the literature that the most targeted daily living skills were personal and domestic skills, with a notable gap in community living skills. The discrepancy may relate to the challenges in operationalizing and measuring outcomes for community living skills, which are inherently more complex and variable than personal and domestic skills (Sparrow et al., 2005). In the literature on teaching community living skills to autistic individuals, the researchers preferred to use different interventions, such as modeling and graduated guidance procedures (Hong et al., 2017). This might be due to the weak feasibility of self-management interventions in such settings. On the other hand, self-management interventions have been found to be effective in improving the community skills of individuals with other disabilities, as evidenced by studies on safety skills (Dixon et al., 2010; Mechling & Stephens, 2009). The lack of investigations concerning autistic individuals raises questions about the allocation of research focus and the potential and broader application of self-management interventions (Reichow et al., 2008). Given the versatility of self-management interventions, researchers should explore incorporating these interventions to teach community living skills to autistic individuals. This shift can help broaden the scope and effectiveness, leading to more comprehensive skill development in this population.

Second, self-monitoring interventions are the most common among self-management strategies in the literature (e.g., Yucesoy-Ozkan & Kartal, 2012; Sulu et al., 2023a, 2023b). Our findings align with the literature that self-monitoring was also the most commonly used self-management strategy to teach daily living skills to autistic individuals. These interventions were used stand-alone in five studies. This may be due to the nature of daily living skills that require a task analysis for effective teaching (Atbasi & Pürsün, 2020), and students self-monitor themselves in order to successfully follow through with skill steps (Pierce & Schreibman, 1994). Although self-monitoring appears to be an effective tool to teach daily living skills, future studies should investigate the efficacy of integrating self-monitoring with other interventions, given the promising outcomes of such collaboratives in the development of a variety of skills (e.g., Dalton et al., 1999), particularly in community living skills.

Third, the majority of the interventions were conducted by the researchers, which is particularly concerning given that daily living skills typically take place in natural environments involving caregivers (Althoff et al., 2019; Gerow et al., 2018). However, caregivers can effectively teach daily living skills to autistic individuals when the necessary support is provided (e.g., Harriage et al., 2016). Caregivers play an important role in teaching daily living skills to individuals with disabilities. Furthermore, all studies were implemented in one-on-one settings. Although certain daily living skills may require one-on-one training, particularly for older participants, the inability of participants to execute these skills may also be linked to their exclusion from general education classrooms where these skills are taught at younger ages. To overcome this challenge, general education teachers can be trained to implement and incorporate self-management interventions in their classrooms (Sulu et al., 2023b; Wood et al., 2002). Additionally, caregivers might be trained for the generalization and maintenance of newly acquired skills. Therefore, future studies should focus on developing self-management intervention packages that include training both teachers and caregivers, ensuring a more holistic approach to teaching daily living skills to autistic individuals.

Fourth, half of the studies did not set a mastery criterion. However, it is crucial for researchers to identify the mastery criteria as an important indicator for assessing the effectiveness of interventions and ensuring the maintenance of the acquired skill (Aydin & Tanious, 2022; Kim et al., 2023; McDougale et al., 2020; Richling et al., 2019; Wong & Fienup, 2022). Evaluating the effectiveness of interventions based on mastery criteria necessitates considering the stages of learning and behavioral fluency (Binder, 1996; Further et al., 2003, Jimenez et al., 2021). Binder (1996) identifed fluency, as a significant aspect of skill mastery, a combination of accuracy and speed. Additional considerations for mastery criteria should include the learner’s efficiency and flexibility in performing the task across various settings and scenarios (Further et al., 2003). Jimenez et al. (2021) expanded on this stages by developing instructional strategies that address each step of learning (i.e., acquisition, fluency, maintenance, and generalization). On the other hand, the present review revealed that the studies with mastery criteria were only based on skill acquisition. This finding is similar to most research that set mastery criteria (Wong et al., 2022). Designing interventions should aim not only at skill acquisition mastery but also to support the learner in achieving a level of performance that facilitates the acquired behavior in everyday life. Therefore, future studies should clearly define and accurately identify mastery criteria to align with the needs of not only skill acquisition but also fluency, maintenance, and generalization.

Fifth, given that the social validity is not a standard in WWC, our descriptive analysis also included this analysis, separetely. Of the 15 studies included in this review, eight did not have any analysis for social validity. However, other quality indicators (e.g., Horner et al., 2005) included social validity analysis as a quality indicator to define an intervention as evidence-based (Aydin et al., 2019). Thus, future studies should conduct social validity assessments to ensure a rigorous analysis and documentation of these criteria, as suggested by Baer et al. (1968) and Wolf (1978).

The positive findings of the current study included the utilization of self-management interventions from participants from diverse categories. Even though our search involved only autistic individuals, many of the participants also had co-occurring intellectual disabilities at varying levels, including severe, moderate, and mild (e.g., Green et al., 2011; Michie et al., 1998). The effectiveness of self-management interventions across a spectrum of cognitive abilities may be attributed to the versatility and applicability of these interventions. This suggests that the benefit of these interventions can be generalized to a broader segment of autistic community. Additionally, the age of the participants showed a variety (e.g., preschool, middle school, adulthood), aligning with the Chia et al. (2018) study. These positive outcomes are important in thinking about the external validity of these interventions and promote access to equitable and inclusive education opportunities for autistic individuals with diverse backgrounds.

Another positive finding of the current study was the diversity and versatility of the self-management interventions being used. Various components are used in the IVs, including written documents, visual-based contents, auditory materials, and prompts. For example, combining self-monitoring with self-instruction techniques, such as videos and task materials, enhances learning by providing clear examples for correct task completion and facilitating self-assessment (e.g., Duttlinger et al., 2013). The use of technology-based aids, such as activity schedules saved on iPhones or online checklists, promotes independence through minimal external prompting (e.g., Bouck et al., 2014). Furthermore, integrating task analysis with self-monitoring, through tools like checklists or picture prompt booklets, supports individuals in following through with skill steps by breaking down complex tasks manageable actions (e.g., Yakubova & Taber-Doughty, 2012). In addition, the combination of self-monitoring with self-reinforcement encourages individuals by allowing them to reward themselves on task completion, thus skill acquisition and self-motivation (e.g., Pierce & Schreibman, 1994). Considering the efficacy of visually-based contents and technology to facilitate the learning of autistic individuals (Aydin & Tekin-İftar, 2020; Cooper et al., 2020; Hrabal et al., 2023), future studies should continue to incorporate these components into the self-management intervention packages and implement across different settings.

Even though the descriptive analyses provided some positive findings, the overall quality of these interventions to demonstrate the efficacy of the self-management interventions appeared to be weak based on WWC standards (Kratochwill et al., 2013). Our analysis revealed that only one study (Yakubova & Taber-Doughty, 2012) met all DSs. One main issue lacks an adequate number of attempts to show the intervention effect. Another weakness was the number of data collected in each phase. Providing adequate attempts to control experimental conditions can strictly enable presenting evidence of how the interventions (i.e., self-management) change the daily living skills of autistic individuals. Therefore, future studies should provide at least three attempts at three distinct times for multiple baseline designs, changing criterion design, and ABAB design, and four different attempts for alternative treatment designs. Additionally, an adequate number of data points (i.e., three or more) should be provided for each condition. Note that the latest WWC guidelines revision now suggests at least six data points for each condition (WWC, 2022).

In effect size calculations, Tau-*U* values were computed for the 14 studies included in this meta-analysis (the Engstrom [2019] study was excluded from effect size calculations due to the lack of comparable baseline data. Actually, Engstrom refers to baseline data in her dissertation. However, when it is reviewed in detail, one can see that the baseline phase is actually an intervention phase of self-monitoring.). The effect sizes for eight of 14 studies were calculated using PCES, as only eight provided mastery criteria. Recall that the effect size analyses were conducted on studies that both met (i.e., with/out reservations) and did not meet the WWC standards. The overall effect size of the studies met (i.e., with/out reservations) WWC standards (*n* = 9) had a very large overall effect size with 0.96 CI_95_ (0.61, 1) for Tau-*U* analyses. Among these 9 studies, 5 also assessed for PCES values and indicated an effective effect size of 1.09. Of the studies did not meet the standards (*n* = 5), the overall effect size was very large with 0.94 CI_95_ (0.50, 1) for Tau-*U* analyses. Among these five studies, three also assessed for PCES values and indicated a moderate effect of 0.90. Therefore, our findings align with the literature, suggesting that there is no difference in estimated effect size when quality indicators are considered (Dowdy, 2018). Regardless of the study quality, the overall effect size of all included studies had a very large overall effect size with 0.93 CI_95_ (0.80, 1) for Tau-*U* analyses. On the other hand, the overall effect size for the PCES values indicated a moderate effect of 0.99. Although Tau-*U* analyses in the individual studies with and without a mastery criterion were the same result as a “very large”, PCES estimates for the individual studies with mastery criteria resulted in “small to high effects”. Considering the methodologically rigorous and effect size values of the included studies in this review, we would identify these interventions as *promising*.

Given the importance of teaching generalization and maintenance of newly acquired skills in the field of ABA, along with the ongoing limitations in related research (Neely et al., 2016; Stokes & Osnes, 1989; Sulu et al., 2023b), an additional systematic meta-analysis was conducted over these two phases. Research indicates that autistic individuals face challenges with stimulus and response generalization; therefore, the successful acquisition of behavior may not indicate that the behavior will be generalized or sustained in different contexts for these individuals (Skinner, 1953; Strokes & Osnes, 1989). However, our search yielded that of the 15 studies included in the review, five were investigated for generalization, and seven studies were collected for maintenance. The findings indicated that generalization and maintenance of daily living skills were very effective for Tau-*U* and considering the moderate to high effect with respect to PCES. Due to the inclusion of a very small number of studies in the analysis, along with the limited number of data points collected across these phases, we are unable to make any conclusion regarding the generalization and maintenance of these interventions. It is, however, evident that planning for these phases is significantly insufficient.

### Limitations and Recommendations

Although our review provided critical insights into the self-management interventions to teach daily living skills, it is not without limitations. First, we did not use the most current WWC standards (WWC, 2022) due to some limitations of the current version, such as minimizing the role of visual analysis, containing subjectivity due to lack of a scoring system, and not possible individual evaluations due to accepting fixed-effect analysis (Maggin et al., 2022). As a result of these criticisms, the recent SCEDs meta-analyses still continue to use previous WWC standards for SCEDs (e.g., Sulu et al., 2024; Tekin-Iftar et al., 2023). Moreover, the literature (cf., Aydin, 2024) offers us several other rubrics for the quality assessment of SCEDs, such as Horner et al. (2005), Reichow et al. (2008), and Council for Exceptional Children (CEC, 2014). Future studies can evaluate and compare the quality of the included studies according to these rubrics. Second, although we used two different effect size analyses to calculate the efficacy of the interventions, different effect size measurements might yield different results. In this regard, there may be a need to see the magnitude of the effect of self-management practices in different effect-size methods. Third, given the limited number of studies included in the review, we did not run additional analyses for moderators. If the body of research about these topics increases in the future, analyses will be needed for moderators of the studies. Last, we did not register our initial search protocol on a foundation such as PROSPERO, Cochrane, Campbell, or Open Science Framework. Systematic review studies should consider registration for their search protocol on a website, which has open access for all other corresponding researchers, to empower the credibility and transparency of research.

## Conclusion

In summary, self-management interventions have been used to improve a variety of the skills of individuals with and without disabilities, but the current review indicated that the investigation of these interventions remained limited for the daily living skills of autistic individuals compared to other skills, such as on-task behaviors (Sulu et al., 2023b). Our search included studies from 1980 to 2023 (expanded to 2024 for gray literature); however, the most recent included two studies were published in 2019, marking a four-year gap without any new published studies in the literature. In addition, within this timeframe, our search yielded a total of 15 published SCEDs studies, yet notably, none of these interventions included community living skills. Even though the overall effect size analysis of 14 studies (for nine studies met WWC standards and five studies did not meet WWC) revealed very large in Tau-*U*, and the overall effect size analysis of eight studies (five met WWC and three did not meet) in PCES revealed a moderate effect, there is a need for new studies with methodological rigor to generalize the efficacy of these interventions. Additionally, these studies should plan to collect generalization and maintenance data for the conducted interventions at the beginning of the study, endeavoring to obtain adequate data.

## Supplementary Information

Below is the link to the electronic supplementary material.

Supplementary file1 (DOCX 70 KB)

## Data Availability

If requested, the corresponding author can provide all documents.

## Code Availability

No code.
