# Supplementary material for: A Meta-Analysis of Self-Management Interventions in Teaching Daily Living Skills to Autistic Individuals
Source: J Autism Dev Disord. 2024 May 6;55(7):2377–92. doi: 10.1007/s10803-024-06355-w (PMC12167301; doi:10.1007/s10803-024-06355-w)
Supplement: Supplementary file 1 — Supplementary file1 (DOCX 70 KB) [file 10803_2024_6355_MOESM1_ESM.docx]

**Supplementary Materials for Entitled**

**“A Meta-Analysis of Self-Management Interventions in Teaching Daily Living Skills to Autistic Individuals”**

**Initial Search Protocol**

**Title:** A meta-analysis of self-management interventions in teaching daily living skills to individuals with autism spectrum disorder (ASD)

**Aim:** To determine descriptive features and effect sizes of self-management interventions for improving the daily living skills of ASD.

**Rationale:** Self-management interventions are crucial for all individuals, including also those with ASD and other developmental disabilities, to independently improve their own skills, such as workplace skills, academics, and daily living skills. Although there are a few systematic reviews and meta-analyses about self-management interventions(e.g., McDougall et al.,2017; Yucesoy-Ozkan & Sonmez, 2011), none are specifically related to teaching daily living skills and also ASD. In this context, there is a need to determine evidence bases of self-management interventions for improving the daily living skills of ASD.

**Research Team:** Three Researchers

**Search Keywords:** “Self-management” OR “self-regulation” OR “self-regulate” OR “self-monitoring” OR “self-recording” OR “self-reinforcement” OR “self-evaluation” OR “self-advocacy” OR “self-observation” OR “self-instruction” OR “empowerment” OR “self-determination” OR “self-control” AND “Autis*” OR “Asperger’s syndrome” OR “PDD-NOS*” AND “daily living skills” OR “self-care” OR “self-help” OR “life-skills” OR “functional skills” OR “leisure skills” OR “recreation skills” OR “living skills” OR “adaptive living” OR “activities of daily living” OR “independen* living” OR “independen* skill” OR “practical skill”.

**Inclusion/Exclusion Criteria:**

We follow the listed inclusion criteria respectively:

1. Have to publish in a peer-reviewed journal in English,
2. Have to use SCEDs,
3. Have to include at least one participant with a diagnosis of ASD in a study,
4. Have to improve the daily living skills,
5. Have to use a self-management strategy as an independent variable,
6. Have to show data in a line graphical format,

*Include when the abovementioned criteria are met; if the other way, exclude.

**Databases:**

ERIC, Academic Seach Ultimate, ScienceDirect, PsycNET (i.e., PsycInfo and PsycArticles), Scopus

**Search Time Limits:**

Between 1980 and 2023

**Steps**

**Step 1: Transfer to Rayyan**

Obtain ris documents from databases.

Combine the obtained ris documents and remove duplicates via Zotero.

Transfer a single ris document to the Rayyan

**Step 2: Evaluate Studies According to Inclusion Criteria on the Rayyan**

Screen title and abstract

Access the full text of possible corresponding articles.

Conduct reference search (i.e., backward search) for the included studies

Conduct cited search on Google Scholar (i.e., forward search) for the included studies

**Step 3: Quality Assessments**

Conduct quality assessments for all included studies according to What Works Clearinghouse Design Standards.

We can use WWC with scores, as in Hong et al. (2016), Neely et al. (2018), and Sulu et al. (2022).

**Step 4: Descriptive Codings**

Conduct descriptive analyses for the studies that met WWC design standards with or without reservations.

Descriptive features can be sought, including participant characteristics, dependent and independent variables, research design, mastery criterion, procedural fidelity, social validity, maintenance, and generalization.

We should create a coding table according to the components of the included studies.

**Step 5: Data Extraction of Graphs for the Effect Size Measures**

Use a free tool called PlotDigitizer Software, which is nearly perfectly reliable and valid for extracting data from single-case graphs (Aydin & Yassikaya, 2022).

**Step 6: Effect Size Measures**

We can calculate the intervention effect sizes through Tau-*U* (Parker et al., 2011) and PCES (Aydin & Tanious, 2022). Both consider data trend at the baseline phase. They differ from each other in terms of calculation approaches. We can see intervention effect sizes more discriminately. However, PCES calculations require mastery/acquisition/performance criteria set in the articles to determine intervention effect sizes (Aydin & Tanious, 2022).

**Step 7: Reliability Analysis**

Conduct reliability analyses for at least 30% of all steps.

**Step 8: Reporting the Research**

Write all processes and report and discuss all findings.

**Task Sharing and Time Framework**

**Step 1: Search and Transfer to Rayyan**

Time: July or August (2023)

Task Personnel: First and third authors

**Step 2: Determining to be Included Articles**

Time: Two months

Task Personnel: Firstly third author, and secondly first author

**Step 3: Quality Assessments**

Time: Three weeks

Task personnel: First and third authors

**Step 4: Descriptive codings**

Time: One month

Task personnel: Firstly third author, and secondly first author. Note that the second author gives ideas for creating a descriptive coding table for the included studies.

**Step 5: Raw Data Extraction from Graphs**

Time: Three weeks

Task personnel: Firstly third author, and secondly first author. The first author gives a brief training to the third author for extracting raw data from graphs through PlotDigitizer Software.

**Step 6: Calculations Intervention Effect Sizes**

Time: Two weeks

Task personnel: First and third authors. The first author gives a brief training to the third author for calculating effect sizes by using the metrics of Tau-U and PCES.

**Step 7: Reliability Analyses**

Time: -

Task personnel: First author

**Step 8: Reporting the Research**

Time: Two months

Task personnel:

Introduction- First and second authors

Discussion- Second and third authors

Method and Results-: First, second, and third authors

Language Edits- Second author

**References**

Aydin, O., & Tanious, R. (2022). Performance criteria‐based effect size (PCES) measurement of single‐case experimental designs: A real‐world data study. *Journal of Applied Behavior Analysis, 55(3),* 891-918.<https://doi.org/10.1002/jaba.928>

Aydin, O., & Yassikaya, M. Y. (2022). Validity and reliability analysis of the PlotDigitizer software program for data extraction from single-case graphs. *Perspectives on Behavior Science*, *45*(1), 239-257.<https://doi.org/10.1007/s40614-021-00284-0>

Hong, E. R., Ganz, J. B., Neely, L., Gerow, S., & Ninci, J. (2016). A review of the quality of primary caregiver-implemented communication intervention research for children with ASD. *Research in Autism Spectrum Disorders, 25*, 122-136. https://doi.org/10.1016/j.rasd.2016.02.005

McDougall, D., Heine, R. C., Wiley, L. A., Sheehey, M. D., Sakanashi, K. K., Cook, B. G., & Cook, L. (2017). Meta‐analysis of behavioral self‐management techniques used by students with disabilities in inclusive settings. *Behavioral Interventions*, *32*(4), 399-417.<https://doi.org/10.1002/bin.1491>.

Neely, L., Garcia, E., Bankston, B., & Green, A. (2018). Generalization and maintenance of functional communication training for individuals with developmental disabilities: A systematic and quality review. *Research in Developmental Disabilities*, *79*, 116-129. <https://doi.org/10.1016/j.ridd.2018.02.002>

Parker, R. I., Vannest, K. J., Davis, J. L., & Sauber, S. B. (2011). Combining nonoverlap and trend for single- case research: Tau-*U*. *Behavior Therapy, 42*(2), 284- 299. https://doi.org/10.1016/j.beth.2010.08.006

Sulu, M. D., Martella, R. C., Grimmet, K., Borosh, A. M., & Erden, E. (2022). Investigating the effects of self-monitoring interventions with students with disabilities on the maintenance and generalization of on-task behavior: A systematic literature review. *Review Journal of Autism and Developmental Disorders, 10*(3), 458-576.<https://doi.org/10.1007/s40489-022-00304-y>.

Yucesoy Ozkan, S., & Sonmez, M. (2011). Examination of single-subject studies conducted on individuals with disabilities by using self-management strategies: A meta-analysis study. *Educational Sciences*: *Theory and Practice*, *11*(2), 809–821.<https://eric.ed.gov/?id=EJ927378>

**Figure S1**

*Data Access Flow Diagram*

Studies screened by full-text

(*n =* 81)

A search of gray literature (1980-2024 February): Proquest and Thesis Global

(*n* = 67)

A search of the databases (1980-2023, August): Academic Search Ultimate, ERIC, Scopus, ScienceDirect, PsycNET

(*n =* 2072)

Duplicates were removed

(*n =* 387)

Studies screened by title and abstract

(*n =* 1752)

Studies were excluded due to not related to the topic or not SCEDs

(*n =* 1671)

**Identification**

**Screening & Eligibility**

Studies were included

(*n =* 10)

A reference search was conducted for the included studies

(*n* = 2)

Google Scholar cited search was conducted for the included studies

(*n* = 1)

**Included**

Review studies were examined for additional possible articles to be included

(*n* = 2)

A total of the included studies for further analyses, including the descriptive analyses, assessments by WWC, and effect size measures

(*n =* 15)

Reasons (*n*)

Not only self-management (37)

Not daily living skills (18)

No an autistic individual (16)

(*n* = 71)

SCEDs: Single case experimental designs; WWC: What Works Clearinghouse

**Table S1**

*WWC standards for SCEDs adapted from Hong et al. (2016) and Neely et al. (2018)*

| **DS1:** When the independent variable was systematically manipulated, it is coded with “1”. A score of "0" was assigned when the independent variable was not subjected to systematic manipulation. |
| --- |
| **DS 2A:** Interobserver agreement is denoted by the code "1." When no interobserver agreement was reported, a rating of "0" was assigned. |
| **DS 2B:** Interobserver agreement was scored as "2" when at least 20% of all sessions and at least 20% of data points for each condition were provided. When interobserver agreement was observed for at least 20% of all sessions but not for at least 20% of the data points for each condition, a "1" was assigned. Studies were scored as "0" if less than 20% of all sessions had documented interobserver agreement. |
| **DS 2C:** If interobserver agreement averaged 80% or above, it was coded as "2", the interrater agreement averaged 80% for some but not all conditions, the rating of "1" was given. Studies averaged less than 80% of interrater agreement was coded with “0”. |
| **DS 3:** Design Standard 3 rated whether the study design included at least three attempts to demonstrate an intervention effect at three different points in time (rating of “2”). Designs that could obtain a rating of “2” included ABAB designs, multiple baseline designs, and changing criterion designs. Alternating treatment designs (ATD) required at least five attempts. A rating of “1” was provided when an ATD had four attempts and all other designs had three attempts with at least one participant, but not all participants. If a study did not include at least three attempts (or 4 for ATD), then the study did not meet this standard and received a rating of “0.” |
| **DS 4:** When a minimum of five data points was provided for each condition, it was coded as "2." When each condition contained at least three data points, a "1" was coded. Any condition with fewer than three data points was recorded as "0" in studies. |
| **Overall Rating:** When all design standards 1–4 were met with the greatest possible rating, the study was coded “meet standards”. If at least one of the design standards 1–4 was coded with “1” where the highest score was 2 and then the study was coded “meet standards with reservations.” An overall rating of “0” indicated that at least one design standard 1–4 was coded with a “0” and considered “did not meet standards.” |

DS: Design standard for single-case experimental designs

**Table S2**

*WWC Standards for G and M phases adapted from Neely et al. (2018)*

| **DS 2A:** If a study specifically identified the collection of interobserver reliability data for maintenance and generalization data, it was rated as “1.” If not, it was rated as “0”. |
| --- |
| **DS 2B:**  A “2” was scored if reliability data were collected for at least 20% of the maintenance data or 20% of the generalization data. If interobserver reliability data was not collected for a minimum of 20% of the maintenance and/or generalization data, it was rated as “0.” |
| **DS 2C:** Maintenance and generalization Design Standard 2C evaluated if the resulting interobserver agreement coefficients met a minimum of 80% agreement or 0.6 kappa. A “1” was scored if the maintenance data or the generalization data met this standard. Less than 80% agreement (0.6 kappa) was rated as “0.” |
| **DS 4:** Maintenance and generalization design standard 4 evaluated whether each phase of the maintenance or generalization data met the minimum criteria for number of data points. A study with more than three data points was rated as “2” and a study with one or two data points was rated as “1. |

DS: Design standard for generalization and maintenance phases in SCEDs

**Table S3**

*Descriptive findings of 15 included studies*

| **Studies** | **Participant Characteristics**  Gender/ Age/ Diagnosis | **Setting(s)**  **and Instructional Arrangement** | **Practitioner** | **DV(s)** | **IV(s)** | **Components of IV(s)** | **Research Design** | **MC** | **TF**  (Ratios and Means) | **SV**  (From Who) | **G**  (What kind?) | **M**  (When? How much data? How many participants?) |
| --- | --- | --- | --- | --- | --- | --- | --- | --- | --- | --- | --- | --- |
| Bereznak et al., 2012 | P1: M; 18; ASD; Mi-ID  P2: M; 15; ASD; Se-ID  P3: M; 15; ASD; Se-ID | School living center and the teacher workroom; one to one | Researcher,  Teacher | Using a washing machine, Making noodles | Self-instruction | Videos  Task Materials | MPD (across skills) | Y | Y  (Ratios: 20>; Means: 91-100%) | N | N | N |
| Bouck et al., 2014 | P1: F; 13; ASD; Mo-ID  P2: M; 15; ASD; Se-ID  P3: F; 15; ASD; Mo-ID | Classroom;  one to one | Researcher | Food preparation skills | Self-monitoring | Checklists, paper/pencil or iPad, least to most prompting procedure, scripts | ATD | N | Y  (Ratios: 20>; Means: 99%) | Y  (Participants and their teacher) | N | Y  (14 weeks after; two data, three participants) |
| Cheung et al., 2016 | P1: F; 8; ASD  P2: M; 10; ASD | Bakery simulation in the classroom; one to one | Researcher | Making purchases of food items in local bakerie | Self-selection,  Self-monitoring | Activity schedules saved on an iPhone | MBD (across participants) | Y | N | Y (External observer) | Y  (settings) | Y  (Every weekday for 2 weeks) |
| Copeland & Hughes, 2000 | P1; M; 15; ASD; Mi-ID | Faculty dining room at the participants’ high school; one to one | Educational assistant | Organizing the table, cleaning under and over the table | Self monitoring | Picture prompt booklet,  Taks analysis | MBD (across participants) | Y | N | N | N | N |
| Duttlinger et al., 2013 | P1: F; 13; ASD; Mi-ID | Participants’ self- contained classroom, the hall immediately adjacent to the classroom, and the bathroom on this hallway; one to one | Teacher | Self-care skills such as brushing teeth and washing hands | Self-monitoring | Picture activity schedules | A-BC-B-A-B withdraval design | Y | Y (Ratios:None; Means: 86-100%) | Y  (Teacher, paraprofessional, speech-language pathologist) | Y  (settings) | N |
| Engstrom, 2019 | P1: F; 20; ASD  P2: M; 19; ASD  P3: M; 20; ASD | Clinic room at the Autism Center of the University; one to one | Clinician | Daily living checklist tasks such as showering, brushing, flossing, food preparation, taking prescribed medication etc. | Self-monitoring | Checklists | MBD (across participants) + Reversal | Y | Y (Ratios: 33%; Means: 100%) | N | N | Y (one week and four weeks after; one data; three participants) |
| Gushanas et al., 2019 | P1: M; 22; ASD | A child care center classroom, community locations, university classrooms and office; one to one | Researcher | Personal hygiene skills | Self-monitoring | Online checklists | MBD (across participants) | N | N | Y  (Participants) | N | N |
| Lee et al., 2007 | P1: F; 17; ASD | Home; one to one | Parent | Nighttime routine | Self-reinforcement,  Self-monitoring | Self-monitoring sheets | ABAB design | N | N | N | N | Y  (18 Months Later, two data, one participants) |
| Lee et al., 2018 | P1: M; 6; ASD, Mi-ID  P2: M; 8; ASD  P3: M; 7; ASD | Kitchen of the house and autism center; one to one | Instructor | Dishwashing | Self monitoring,  Self recording | Dishwashing task analysis booklet, self- management recording form | MPD (across participants) | Y | N | Y  (Parents) | N | Y  (1 week after, two data, all participants) |
| Mays & Heflin, 2011 | P1: M; 6; ASD; Mo-ID  P2: M; 11; ASD; Mo-ID  P3: F; 7; ASD; Mo-ID  P4: F; 11; ASD; Mo-ID | Restroom; one to one | Teacher | Tooth brushing, hand washing | Self-instruction | Cassette tape player | MBD (across participants) | N | Y (Ratios:None; Means: 100%) | Y  (Parents) | N | N |
| Mechling & Stephens, 2009 | P1: M; 22; ASD; Mo-ID | Kitchen; one to one | Researcher | Cooking | Self-instruction | Picture based cookbook, DVD player, | ATD | N | Y (Ratios:None; Means: 86-100%) | N | N | N |
| Parker & Kamps, 2011 | P1: M; 9; HFA  P2: F; 9; HFA | Home kitchen, school, restaurant; one to one | Para-professional | Cooking, restaurant activities | Self-monitoring, self-recording | Task analysis | MPD (across skills) | Y | N | N | N | N |
| Pierce & Schreibman, 1994 | P1: M; 8; ASD; Mo-ID  P2: M; 9; ASD; Se-ID  P3: M; 6; ASD; Mo-ID | Clinic room; one to one | Therapist | Setting the table, making a drink, making the bed | Self-monitoring,  Self-reinforcement | Picture prompts, task analysis | MPD (across skills) | Y | N | N | Y  (settings) | Y  (2 months after, two data, all participants) |
| Stokes et al., 2004 | P1: M; 36 to 38 (not specified); ASD | Bathroom; one to one | Researcher | Personal hygiene skills | Self-monitoring  Self-evaluation | Task analysis | AB design | Y | N | N | Y  (Settings) | Y  (one and 9 months after, two data) |
| Yakubova & Taber-Doughty, 2012 | P1:M; 16; HFA; Mi-ID  P2:M; 19; HFA; Mi-ID | Classroom that included a full kitchen, dining area, and a bathroom; one to one | Researcher | Cleaning the mirror, sink and floor | Self- monitoring | Video modeling clips, self-monitoring checklists, SMART notebook, electronic folders | MPD (across participants) | N | Y (Ratios:  30 >; Mean:100%) | Y  (Teacher, participants) | Y  (settings) | N |
| Total Autistic Participants | 20 M ASD;  9 F ASD;  2 M HFA |  |  |  |  |  |  |  |  |  |  |  |

P: Participants; F: Female, M: Male, ASD: Autism Spectrum Disorder, HFA: High Functioning Autism, Mo: Moderate; Mi: Mild; Se: Severe; ID: Intellectual Disability; DV(s): Dependent Variable(s); IV(s): Independent Variable(s); ATD: Alternating Treatment Design; MPD: Multiple Probe Design, MBD: Multiple Baseline Design; MC: Mastery Criterion; Y: Yes; N: No; IOA: Interobserver Agreement; TF: Treatment Fidelity; SV: Social Validity; G: Generalization; M: Maintenance.

**Table S4**

*Quality Assessment Considering WWC Standards for SCEDs*

| **Study** | **Overall** | **DS#1** | **DS#2A** | **DS#2B** | **DS#2C** | **DS#3** | **DS#4** | **Evaluation** |
| --- | --- | --- | --- | --- | --- | --- | --- | --- |
| Bereznak et. al., 2012 | 1 | 1 | 1 | 2 | 2 | 2 | 1 | MS-R |
| Bouck et al., 2014 | 1 | 1 | 1 | 2 | 1 | 2 | 2 | MS-R |
| Cheung et al., 2016 | 0 | 1 | 1 | 1 | 2 | 0 | 1 | Non-MS |
| Copeland & Hughes, 2000 | 0 | 1 | 1 | 1 | 1 | 0 | 2 | Non-MS |
| Duttlinger et al., 2013 | 1 | 1 | 1 | 2 | 2 | 2 | 1 | MS-R |
| Engstrom, 2019 | 0 | 1 | 1 | 2 | 2 | 0 | 1 | Non-MS |
| Gushanas & Thompson, 2019 | 1 | 1 | 1 | 2 | 1 | 2 | 2 | MS-R |
| Lee et al., 2007 | 0 | 1 | 0 | 0 | 0 | 2 | 0 | Non-MS |
| Lee et al., 2018 | 1 | 1 | 1 | 1 | 1 | 2 | 1 | MS-R |
| Mays & Heflin, 2011 | 1 | 1 | 1 | 1 | 2 | 2 | 2 | MS-R |
| Mechling & Stephens, 2009 | 0 | 1 | 1 | 2 | 2 | 0 | 1 | Non-MS |
| Parker & Kamps, 2011 | 1 | 1 | 1 | 2 | 2 | 2 | 1 | MS-R |
| Pierce & Schreibman, 1994 | 1 | 1 | 1 | 2 | 1 | 2 | 1 | MS-R |
| Stokes et al., 2004 | 0 | 1 | 1 | 1 | 1 | 0 | 2 | Non-MS |
| Yakubova & Taber-Doughty, 2012 | 2 | 1 | 1 | 2 | 2 | 2 | 2 | MS |

DS= Design standard for SCEDs; MS: meet standards; MS-R: meet standards with reservations; Non-MS: not meet standards

**Table S5**

*Quality Assessment and Effect Size Measures for Generalization and Maintenance Data*

| **Study** | **DS#2A**  **G/M** | **DS#2B**  **G/M** | **DS#2C**  **G/M** | **DS#4**  **G/M** | **Effect Sizes** | |
| --- | --- | --- | --- | --- | --- | --- |
|  |  |  |  |  | **G/M**  **Tau-*U*_weighted_** | **G/M**  **PCES_weighted_** |
| Bouck et al., 2014-iPad | -/0 | -/0 | -/0 | -/1 | -/.95 CI_95_ (.21, 1) | - |
| *Cheung et al., 2016 | 0/0 | 0/0 | 0/0 | 2/2 | .92 CI_95_ (.41; 1)/1 CI_95_ (.57; 1) | 1.05/1.25 |
| Duttlinger et al., 2013 | 1/- | 2/- | 2/- | 1/- | 1 CI_95_ (.1, 1)/- | 1.6/- |
| *Engstrom, 2019 | -/0 | -/0 | -/0 | -/1 | Nan | Nan |
| *Lee et al., 2007 | -/0 | -/0 | -/0 | -/0 | -/1 CI_95_ (.20; 1) | - |
| Lee et al., 2018 | -/1 | -/2 | -/2 | -/1 | -/1 CI_95_ (.36, 1) | -/.97 |
| Pierce & Schreibman, 1994 | 1/1 | 2/2 | 2/2 | 1/1 | 1 CI_95_ (.55, 1) /.94 CI_95_ (.60, 1) | .94/.89 |
| *Stokes et al., 2004 | 0/0 | 0/0 | 0/0 | 2/2 | 1 CI_95_ (.38; 1) /1 CI_95_ (.31; 1) | 1/1 |
| Yakubova & Taber-Doughty, 2012 | 1/- | 2/- | 2/- | 2/- | 1 CI_95_ (.64, 1)/- | - |

Asterisks show the studies that do not meet WWC standards with or without reservations. DS= Design Standard for SCEDs, G = Generalization, M = Maintenance; Nan: It is not applicable because there is no comparable baseline data without any treatment
